# PTPN22 is associated with susceptibility to psoriatic arthritis but not psoriasis: evidence for a further PsA-specific risk locus

**DOI:** 10.1136/annrheumdis-2014-207187

**Published:** 2015-04-28

**Authors:** John Bowes, Sabine Loehr, Ashley Budu-Aggrey, Steffen Uebe, Ian N Bruce, Marie Feletar, Helena Marzo-Ortega, Philip Helliwell, Anthony W Ryan, David Kane, Eleanor Korendowych, Gerd-Marie Alenius, Emiliano Giardina, Jonathan Packham, Ross McManus, Oliver FitzGerald, Matthew A Brown, Frank Behrens, Harald Burkhardt, Neil McHugh, Ulrike Huffmeier, Pauline Ho, Andre Reis, Anne Barton

**Affiliations:** 1 Arthritis Research UK Centre for Epidemiology, Centre for Musculoskeletal Research, Institute for Inflammation and Repair, Manchester Academic Health Science Centre, The University of Manchester, Manchester, UK; 2 Institute of Human Genetics, University of Erlangen-Nuremberg, Erlangen, Germany; 3 NIHR Manchester Musculoskeletal Biomedical Research Unit, Central Manchester University Hospitals NHS Foundation Trust, Manchester Academic Health Science Centre, Manchester, UK; 4 The Kellgren Centre for Rheumatology, Central Manchester Foundation Trust, NIHR Manchester Biomedical Research Centre, Manchester, UK; 5 Monash University, Melbourne, Victoria, Australia; 6 NIHR-Leeds Musculoskeletal Biomedical Research Unit, Leeds Institute of Molecular Medicine, University of Leeds, Leeds, UK; 7 Department of Clinical Medicine, Institute of Molecular Medicine, Trinity College Dublin, Dublin, Ireland; 8 Adelaide and Meath Hospital and Trinity College Dublin, Dublin, Ireland; 9 Royal National Hospital for Rheumatic Diseases and Department Pharmacy and Pharmacology, University of Bath, Bath, UK; 10 Department of Public Health and Clinical Medicine, Rheumatology, University Hospital, Umeå, Sweden; 11 Department of Biopathology, Centre of Excellence for Genomic Risk Assessment in Multifactorial and Complex Diseases, School of Medicine, University of Rome ‘Tor Vergata’ and Fondazione PTV ‘Policlinico Tor Vergata’, Rome, Italy; 12 Rheumatology Department, Haywood Hospital, Health Services Research Unit, Institute of Science and Technology in Medicine, Keele University; 13 Department of Rheumatology, St. Vincent's University Hospital, UCD School of Medicine and Medical Sciences and Conway Institute of Biomolecular and Biomedical Research, University College Dublin, Dublin, Ireland; 14 The University of Queensland Diamantina Institute, Translational Research Institute, Princess Alexandra Hospital, Woolloongabba, Brisbane, Queensland, Australia; 15 Division of Rheumatology and Fraunhofer IME-Project-Group Translational Medicine and Pharmacology, Goethe University, Frankfurt, Germany

## Abstract

**Objectives:**

Psoriatic arthritis (PsA) is a chronic inflammatory arthritis associated with psoriasis; it has a higher estimated genetic component than psoriasis alone, however most genetic susceptibility loci identified for PsA to date are also shared with psoriasis. Here we attempt to validate novel single nucleotide polymorphisms selected from our recent PsA Immunochip study and determine specificity to PsA.

**Methods:**

A total of 15 single nucleotide polymorphisms were selected (P_Immunochip_ <1×10^−4^) for validation genotyping in 1177 cases and 2155 controls using TaqMan. Meta-analysis of Immunochip and validation data sets consisted of 3139 PsA cases and 11 078 controls. Novel PsA susceptibility loci were compared with data from two large psoriasis studies (WTCCC2 and Immunochip) to determine PsA specificity.

**Results:**

We found genome-wide significant association to rs2476601, mapping to *PTPN22* (p=1.49×10^−9^, OR=1.32), but no evidence for association in the psoriasis cohort (p=0.34) and the effect estimates were significantly different between PsA and psoriasis (p=3.2×10^−4^). Additionally, we found genome-wide significant association to the previously reported psoriasis risk loci; *NOS2* (rs4795067, p=5.27×10^−9^).

**Conclusions:**

For the first time, we report genome-wide significant association of *PTPN22* (rs2476601) to PsA susceptibility, but no evidence for association to psoriasis.

## Introduction

Psoriatic arthritis (PsA) is a chronic inflammatory arthritis associated with psoriasis; in UK populations the prevalence rate of PsA in patients with psoriasis is estimated to be 14%.^1^ While psoriasis has a serious impact on the patient's quality of life, those suffering from PsA have been found to have a lower quality of life than psoriasis alone.^2^

PsA is a complex disease with environmental and genetic risk factors contributing to the overall liability. The genetic factors contributing to the susceptibility of PsA are not fully understood, but PsA is estimated to have a larger genetic component than psoriasis.^3^ This suggests a substantial difference in the genetic architecture of the two diseases with a heavier genetic burden for PsA. Many of the genetic risk loci reported as associated with PsA susceptibility are shared with psoriasis indicating the importance of pleiotropic effects within shared molecular pathways mediated by the presence of cutaneous psoriasis in both phenotypes. Recent studies have identified PsA-specific loci that begin to explain this increased burden; the presence of glutamic acid at the amino acid position 45 in HLA-B has been shown to be a risk factor for PsA in a psoriasis cohort and our recent Immunochip study confirmed the independent *HLA-B* association.^4^ In addition, we reported evidence for a PsA-specific risk locus at chromosome 5q31 and distinct PsA variants at the *IL23R* locus.^5^

The aim of the current study was to test the loci at suggestive levels of significance in our recent Immunochip analysis to identify novel PsA loci in a large collection of PsA cases and controls collected from the UK, Ireland, Germany, Australia, Sweden and Italy.

## Methods

### Samples

All samples included in this study were of European ancestry and provided written informed consent. Summary statistics and genotype data were available from the PsA Immunochip study comprising 1962 cases and 8923 controls.^5^ In addition genotype data was available for the psoriasis Wellcome Trust Case Control Consortium 2 (WTCCC2) study which contained 1784 psoriasis samples following exclusion of known PsA samples and 5175 controls.^6^ A total of 1352 PsA case and 2164 control DNA samples, independent of those tested as part of the Immunochip study, were available for genotyping collected from Germany (cases=508, controls=920), Sweden (cases=417, controls=1079) and Italy (cases=427, controls=165). A description of clinical characteristics for the three cohorts is provided in online supplementary table S1. Data for a total of 3139 PsA cases and 11 078 controls were available for this study following quality control.

### SNP selection and genotyping

A total 15 single nucleotide polymorphisms (SNPs) were selected from the Immunochip study based on a significance threshold of p<1×10−4.^7^ Genotyping was performed using the Life Technologies TaqMan chemistry on the QuantStudio genotyping platform at the University of Erlangen, Germany. Sample and SNPs with low call rates (<0.9) were excluded prior to analysis. All genotype cluster plots were manually reviewed and SNPs were screened for deviation from Hardy-Weinberg equilibrium in control samples (Bonferroni corrected p<3.3×10^−3^).

### Statistical analysis

Association testing was performed using logistic regression implemented in PLINK and meta-analysis of Immunochip and validation summary statistics was performed, weighting SNPs by inverse-variance and assuming fixed effects, using the software package METAL.

For loci not previously reported as being associated with psoriasis susceptibility we investigated PsA-specificity using two large psoriasis studies. First, we tested association to psoriasis using genotype data from WTCCC2 and association summary statistics from the largest psoriasis study to date, consisting of 10 588 psoriasis cases and 22 806 controls,^8^ from ImmunoBase (http://www.immunobase.org). Second, we compared effect estimates in PsA to psoriasis using multinomial logistic regression using genotype data for PsA cases and controls from Immunochip and psoriasis genotype data from WTCCC2 performed in Stata. Finally, we directly compared PsA and psoriasis genotypes, with PsA coded as cases and psoriasis coded as controls. Sex differentiated associations were investigated by analysing men and women separately and comparing differences in effect estimates using Cochrane's Q statistic using Immunochip genotype data.

To control for phenotype misclassification with rheumatoid arthritis (RA), we included a genetic risk score (GRS) comprised of the 41 non-HLA RA susceptibility SNPs reported in the RA Immunochip study, weighted by odds ratio (OR), as a covariate and recalculated the PsA Immunochip summary statistics.^9^ ^10^

## Results

**Figure 1:**
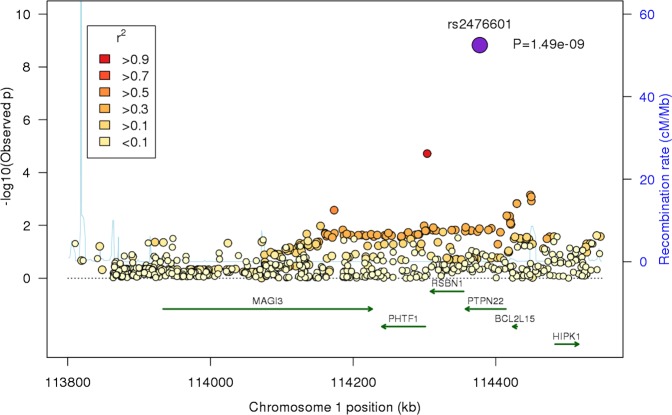
Regional association plots for the *PTPN22* locus for PsA Immunochip data and meta-analysis of rs2476601. The x-axis represents chromosomal position and gene location. The first y-axis represents –log_10_ of the observed p value from logistic regression, secondary y-axis represents estimated recombination rates (cM/Mb). Circles represent genotyped single nucleotide polymorphisms (SNPs), colour of the circle represents linkage disequilibrium (r^2^) with the index SNP (purple circle). kb, kilobase; cM, centimorgan; Mb, megabase.

**Figure 2:**
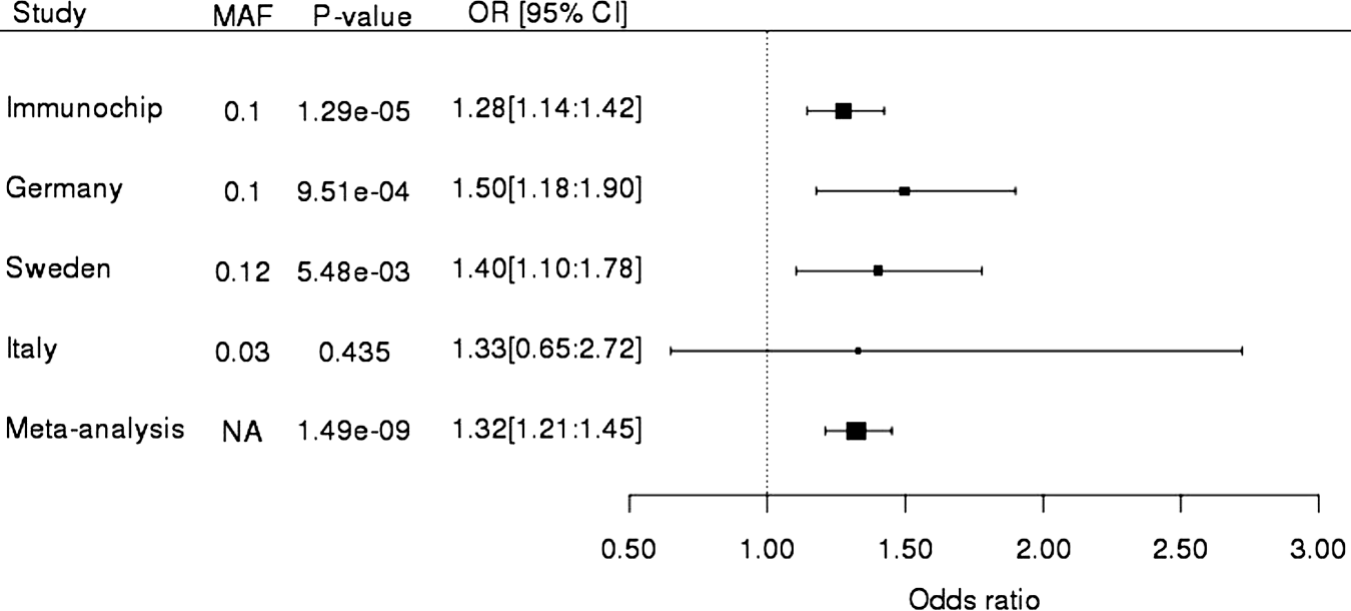
Forest plot of effect estimates for rs2476601 from the Immunochip, validation and meta-analysis. Rows are labelled by study group and include MAF, p values, ORs and 95% CIs. Reported MAF is estimated from control group, for Immunochip cohort this is estimated from UK controls. CI, confidence interval; minor allele frequency; OR, odds ratio.

Following quality control of the validation genotype data a total of 13 SNPs for 1177 cases and 2155 controls was available for analysis. Meta-analysis of the validation samples with Immunochip data resulted in a combined data set of 3139 PsA cases and 11 078 controls. We identified genome-wide significance to two loci; *NOS2* (rs4795067, p=5.27×10^−9^) and *PTPN22* (rs2476601, p=1.49×10^−9^) (table 1). Association to *NOS2* has previously been reported to psoriasis; however no such association has been made to *PTPN22* (figures 1 and 2). Interestingly we observe a higher effect estimate for rs2476601 in men compared with women (1.31 vs 1.22, respectively) as previously reported for this SNP in PsA, however this difference is not statistically significant (Q=0.52). We also observe a much lower minor allele frequency for rs2476601 in the Italian population which is consistent with previous studies demonstrating a North-East to South-West gradient for minor allele frequency (MAF) across continental Europe.^11^

**Table 1:** Summary statistics for Immunochip, validation and meta-analysis of selected SNPs

rs	chr	bp	Gene	Risk/non-risk	Immunochip (cases=1962, controls=8923)			Validation (cases=1177, controls=2155)		Meta-analysis (cases=3139, controls=11 078)			
					RAF	p Value	OR	p Value	OR	p Value	OR	I2	Q
rs2476601	1	114 377 568	PTPN22	A/G	0.10	1.29E-05	1.28	1.28E-05	1.44	1.49E-09	1.32	0	0.65
rs4795067	17	26 106 675	NOS2	G/A	0.34	1.94E-07	1.21	7.42E-03	1.25	5.27E-09	1.22	0	0.75
rs984971	2	163 224 521	KCNH7	G/A	0.36	3.62E-06	0.84	0.02	0.87	2.29E-07	0.85	0	0.61
rs1306395	2	61 076 272	LINC01185	C/T	0.43	2.99E-05	0.86	0.04	0.88	3.43E-06	0.87	0	0.85
rs7552167	1	24 518 643	IFNLR1	A/G	0.14	1.53E-05	0.79	0.10	0.88	7.36E-06	0.82	35.6	0.20
rs8106664	19	10 728 030	SLC44A2	G/T	0.23	3.28E-06	0.81	0.13	0.89	1.67E-06	0.83	0	0.52
rs2392581	7	38 573 234	AMPH	G/A	0.42	6.90E-05	0.87	0.17	0.93	4.42E-05	0.88	51.7	0.10
rs8103241	19	13 122 612	NFIX	G/A	0.46	9.08E-05	0.87	0.19	0.92	5.41E-05	0.88	0	0.52
rs1133071	9	32 455 674	DDX58	C/T	0.30	3.36E-05	1.17	0.20	1.09	2.49E-05	1.15	64.9	0.06
rs6713082	2	62 516 544	B3GNT2	A/C	0.24	4.59E-05	1.18	0.46	1.05	9.44E-05	1.15	71.7	0.03
rs2298428	22	21 982 892	YDJC	T/C	0.18	4.38E-05	1.20	0.56	1.04	2.35E-04	1.14	66.8	0.03
rs8016947	14	35 832 666	NFKBIA	T/G	0.44	9.65E-05	0.87	0.73	1.02	1.49E-03	0.91	70.1	0.04
rs7895120	10	129 064 193	DOCK1	T/C	0.14	5.29E-05	0.80	0.87	1.01	1.44E-03	0.87	62.9	0.04

bp, base position; chr, chromosome; I^2^, heterogeneity index for ORs; Q, Cochrane's Q statistic for heterogeneity of ORs; RAF, risk allele frequency;

As SNPs at the *PTPN22* locus have not previously been reported to be associated to psoriasis susceptibility we investigated this further in two large psoriasis data sets. First we analysed genotyped data from the WTCCC2 psoriasis study, excluding known PsA samples (cases n=1784, controls n=5175), for rs2476601 and found no evidence for association (p=0.34). Second we searched summary statistics from the largest psoriasis study to date (cases n=10 588, controls n=22 806) using the ImmunoBase database and again found no evidence for association of rs2476601 to psoriasis susceptibility (p=0.49). Using genotype data from the PsA Immunochip study and WTCCC2 we directly compared the effect estimates for rs2476601 in PsA and psoriasis using multinomial logistic regression and we found the estimates to be significantly different (p=3.2×10^−4^). A direct comparison of genotypes for PsA (n=1962) and psoriasis (n=1784) found significant association to an increased risk of PsA (p=4.4×10^−4^, OR=1.3).

Given that rs2476601 is a genetic risk factor for RA we were concerned that the observed p value in the discovery study was a false positive due to phenotype misclassification caused by the presence of unidentified RA samples in the case cohort. However, we found the association to rs22476601 in the PsA Immunochip data was unaffected by the inclusion of the RA-GRS (p=1.29×10^−5^ vs P_GRS_=1.30×10^−5^).

## Discussion

In this study we present evidence for association of rs2476601 to susceptibility of PsA exceeding the threshold recognised as genome-wide significant (p<5×10^−8^) for the first time. In addition we used genotype data and summary statistics from two large psoriasis studies to demonstrate that this locus is differentially associated to PsA and not psoriasis per se. We also confirm association of PsA with a previously reported psoriasis locus, *NOS2*, bringing the total number of confirmed, genome-wide significant, PsA loci to 10 including 4 that are PsA-specific (*HLA-B*, chromosome 5q31, PsA-specific variants within *IL23R* and now *PTPN22*). Studies have shown that PTPN22 is a potent inhibitor of T cell activation and it is possible that the effect may differ between T cell subpopulations.^12^ For example we have shown that CD8+ T cells are important for PsA, while this has not been reported in psoriasis.^5^

Strengths of the current study include the large sample sizes used, which allowed us to confirm association at accepted genome-wide thresholds. Previous studies of this locus in PsA have been limited by small sample size; results have either shown weak evidence for association;^13^ ^14^ weak association in men only^15^ or no evidence for association at all.^16^ Indeed, our previous attempts to investigate rs2476601 and PsA susceptibility failed to find any evidence of association.^17^ This previous study had approximately 60% power to detect an effect of the size estimated in the current study. The absence of association for rs2476601 in the Italian cohort of this study is attributed to reduced power due to the much lower MAF (figure 2). Previous investigations of the rs2476601 PTPN22 variant with psoriasis have consistently reported no evidence for association,^18^ ^19^ but some have found association to other variants in the region, for example to rs3789604 (*RSBN1*) or haplotypes spanning *PTPN22.*^20^ ^21^ However, in the largest psoriasis genetic association study performed to date, no association was detected to either rs2476601 or rs3789604 (p=0.49 and p=1.00, respectively).^8^ Indeed, a direct comparison of psoriasis and PsA confirmed that the rs2476601 association is PsA-specific, making it the fourth such locus to be identified.

In contrast to the previous reports, the study presented here is performed in a large cohort of 3139 cases and 11 078 controls, includes independent validation and, for the first time, reports confirmed association with susceptibility to PsA exceeding genome-wide significance (p=1.49×10^−9^). The identification of PsA-specific loci is vital in terms of understanding the different pathways involved, which may require different treatments, and for future screening strategies to identify subjects at risk of developing PsA in patients with psoriasis.

The SNP, rs2476601, has been found to be associated with multiple autoimmune diseases including RA, where the association is predominantly found in anti-citrullinated protein antibody (ACPA)-positive subjects, although association in the ACPA-negative subgroup has been reported.^22^ One possibility, therefore, is that the association with PsA could be due to the inclusion of patients with RA and coincidental psoriasis in the PsA cohort. Unfortunately, ACPA or rheumatoid factor status was not available for many samples. A strength of the current study, however, is that we used a GRS of known RA loci, which has been previously shown to adequately control for potential phenotype misclassification, to explore this possible confounder and found that the association with PsA remained statistically significant even after this adjustment.^10^

In conclusion we report for the first time genome-wide significant association of the rs2476601 variant in the *PTPN22* gene with susceptibility to PsA consistent with reports in many other autoimmune diseases. In addition, we use genotype data from a large psoriasis study to demonstrate that rs2476601 is differentially associated to PsA and not psoriasis.

## Supplementary Material

Web table
