# Supplementary material for: PTPN22 is associated with susceptibility to psoriatic arthritis but not psoriasis: evidence for a further PsA-specific risk locus
Source: Ann Rheum Dis. 2015 Apr 28;74(10):1882–5. doi: 10.1136/annrheumdis-2014-207187 (PMC4602265; doi:10.1136/annrheumdis-2014-207187)
Supplement: Web table [file annrheumdis-2014-207187-s1.pdf]

	German	Swedish	Italian
Age (mean±SD)	58 (11.6)	55.2 (13)	57 (13.2)
Gender (% female)	47.4	51.4	38
CASPAR criteria	100%	84%	100%
Rheumatoid factor negative	>98%	84.5	not available
Disease duration year (mean±SD)	not available	16 (12)	not available

Table S1: Clinical characteristics of the three validation cohorts.
